# Visual and surgical outcomes of traumatic cataract surgery in India

**DOI:** 10.6026/973206300214510

**Published:** 2025-12-15

**Authors:** Rohit Kumar Mahato, Sunil Kumar, Mousmi Anand, Deepankar Deepankar, Neda Rahman

**Affiliations:** 1 Regional Institute of Ophthalmology, Rajendra Institute of Medical Sciences, Ranchi, Jharkhand, India

**Keywords:** Traumatic cataract, phacoemulsification, visual outcomes, ocular trauma, ocular trauma

## Abstract

Traumatic cataract is a major cause of visual impairment, particularly in regions with high rates of ocular trauma. Therefore, it is of interest to evaluate visual and surgical outcomes following phacoemulsification in 62 patients with traumatic cataract at a tertiary center in Jharkhand. Most patients presented with poor preoperative vision and blunt trauma was more common than penetrating injuries. At 60 days postoperatively, 88.7% achieved 6/18 vision or better, with cystoid macular edema being the most frequent complication. Traumatic cataract surgery significantly improves visual outcomes, supporting early intervention and proper surgical technique for optimal results.

## Background:

Traumatic cataract represents one of the leading causes of visual impairment worldwide, constituting a significant proportion of cataract cases, particularly in low-resource settings where occupational and environmental hazards are prevalent [1]. Unlike age-related cataracts, traumatic cataracts present unique challenges due to their association with additional ocular injuries, varying severity of trauma and potential for intraoperative complications [2]. The impact of traumatic cataracts extends beyond visual impairment, affecting individuals in their productive years and leading to substantial socioeconomic consequences. Ocular trauma is a major public health concern, frequently occurring in regions where agricultural, industrial and road traffic accidents are common. The pathophysiology of traumatic cataract involves mechanical damage to the crystalline lens, leading to disruption of lens fibers and progressive opacification [3]. The mechanism of cataract formation varies depending on the type and severity of trauma, with blunt trauma causing rosette cataracts and lens dislocation, while penetrating trauma results in immediate lens opacity requiring urgent surgical intervention [4]. The large rural population of Jharkhand, a state in eastern India, is predominantly engaged in manual labor, mining and other high-risk occupations, resulting in a significant burden of ocular trauma. The accessibility and availability of specialized ophthalmic care in tertiary care centers play a crucial role in managing such cases [5]. However, research on the surgical and visual outcomes of traumatic cataract cases in this region remains limited, creating a knowledge gap that needs to be addressed. Surgical management of traumatic cataracts is inherently challenging due to associated complications such as corneal scarring, zonular instability, lens capsule rupture and secondary inflammation [6]. The outcomes of cataract surgery in these cases depend on various factors, including the presence of associated ocular injuries, extent of trauma and timing of surgical intervention. Despite recent advancements in surgical techniques, the visual prognosis in traumatic cataract patients remains unpredictable and requires careful evaluation [7]. The clinical significance of traumatic cataracts extends beyond sudden visual loss, as they predominantly affect younger individuals whose livelihood depends on good vision. The psychological impact, loss of employment opportunities and reduced social mobility associated with traumatic cataracts can be profound [8]. Surgical intervention for traumatic cataracts is often more complex than routine cataract surgery, requiring careful consideration of factors such as capsular support, extent of lens opacification and risk of intraoperative complications. Postoperative visual outcomes in traumatic cataract patients are highly variable, with some patients achieving significant visual improvement while others experience residual visual impairment due to pre-existing ocular damage [9]. Secondary complications such as glaucoma, cystoid macular edema, or corneal decompensation may further impact long-term visual rehabilitation. Understanding these outcomes is essential for improving surgical techniques, optimizing postoperative management and setting realistic patient expectations. Therefore, it is of interest to address the research gap by providing comprehensive evaluation of visual and surgical outcomes following cataract surgery in patients with traumatic cataracts at a tertiary care center in Jharkhand.

## Materials and Methods:

## Study design:

This study was designed as a prospective hospital-based observational study conducted at the Regional Institute of Ophthalmology, Rajendra Institute of Medical Sciences, Ranchi, which serves as a tertiary referral centre providing specialized ophthalmic care to the population of Jharkhand and surrounding regions.

## Study population:

The study population comprised patients diagnosed with traumatic cataract who attended the ophthalmology department during the study period from July 2023 to December 2024. All patients who met the inclusion criteria and provided informed consent were enrolled in the study. The sample size was calculated based on clinically relevant risk ratio for predicting visual acuity outcomes at 60 days post-cataract surgery in traumatic cataract patients, considering a 40% proportion of events in the non-exposed group, alpha level of 5%, 80% power and 10% dropout rate, resulting in an estimated sample size of 62 subjects.

## Inclusion and exclusion criteria:

Patients diagnosed with traumatic cataract who were planned for cataract surgery during the study period were included in the study. The exclusion criteria comprised patients with central corneal scarring, posterior segment pathologies such as vitreous haemorrhage, retinal detachment, or choroidal detachment, patients with intraocular foreign body and patients unwilling to participate in the study. These exclusion criteria were applied to ensure that the study population represented cases where surgical outcomes could be primarily attributed to traumatic cataract rather than other confounding ocular pathologies.

## Methodology:

A detailed history was recorded for each patient, including demographic data, mode of ocular trauma, affected eye, causative agent and type of trauma. Each patient underwent comprehensive general and systemic examination followed by detailed ophthalmic evaluation. The ophthalmic assessment included best-corrected visual acuity assessment using Snellen's chart, Landolt's C test, or E chart depending on patient cooperation and capability. Slit-lamp biomicroscopy was performed to evaluate anterior segment abnormalities including corneal involvement, anterior chamber reaction, synechiae and capsular status. Dilated fundus examination was conducted using indirect ophthalmoscopy with +20D Volk lens and direct ophthalmoscopy for detailed macular and optic nerve assessment. B-scan ultrasonography was performed in cases with media opacities to assess retinal and vitreous status, while routine X-ray orbit was performed when indicated to detect intraocular foreign bodies or orbital fractures. A-scan biometry and keratometry were performed for intraocular lens power calculation using the SRK/T formula, with IOL power of the contralateral eye considered in patients with corneal scarring. All patients underwent phacoemulsification with posterior chamber intraocular lens implantation as the preferred surgical technique. Adult patients were operated under peribulbar anaesthesia, while uncooperative patients and children underwent surgery under general anaesthesia. The surgical approach was modified based on intraoperative findings, including capsular support and integrity of zonules. Postoperative follow-up evaluations were conducted on days 1, 7, 21, 45 and 60, with comprehensive ophthalmic examination including visual acuity assessment, intraocular pressure measurement, slit-lamp examination of anterior segment, fundus examination and evaluation of IOL position and postoperative inflammation. Any complications were identified and managed appropriately.

## Statistical analysis:

All relevant clinical data were recorded systematically and analyzed using SPSS version 22 and STATA version 13. Descriptive statistics were used to represent baseline and demographic characteristics of study participants. Categorical data were presented using numbers and percentages, while continuous data were represented using mean and standard deviation for normally distributed data and median with interquartile range for non-normally distributed data. Chi-square test was used for categorical data comparison, Student's t-test for normally distributed continuous data and Mann-Whitney U test for non-normally distributed continuous data. Logistic regression analysis was performed to identify predictors of binary outcomes, with clinically relevant and statistically significant variables considered for multivariable analysis. A p-value of less than 0.05 was considered statistically significant.

## Ethical considerations:

Ethical clearance was obtained from the Institutional Ethics Committee of Rajendra Institute of Medical Sciences, Ranchi, prior to commencement of the study. Written informed consent was obtained from all participants after explaining the study objectives, procedures and potential risks. For pediatric patients, consent was obtained from parents or guardians. Patient confidentiality was maintained throughout the study period and all data were anonymized for analysis purposes.

## Results:

The study included 62 patients with traumatic cataract, with age ranging from 10 to 69 years and a mean age of 39.5 years (standard deviation 15.6). The demographic distribution revealed a predominance of male patients, accounting for 70% of the study population, with males having a mean age of 41.2 years compared to 35.6 years for females. The involvement of right and left eyes was equally distributed, with 31 patients (50%) having right eye involvement and 31 patients (50%) having left eye involvement, as shown in Table 1 (see PDF). Analysis of trauma types revealed that blunt trauma was more common than penetrating trauma, with 37 patients (59.67%) experiencing blunt trauma and 25 patients (40.32%) having penetrating trauma. This distribution suggests that workplace accidents, falls and similar mechanisms causing blunt force injuries are leading causes of traumatic cataracts in this population. Preoperative visual acuity assessment demonstrated severe visual impairment in the majority of patients, with 19 patients (30.64%) presenting with counting fingers vision and 25 patients (40.32%) having 6/60 vision. Additionally, 12 patients (19.35%) had 6/36 vision and 6 patients (9.68%) had 6/24 vision, indicating that most patients had vision insufficient for routine daily activities prior to surgical intervention. All cases were managed using phacoemulsification technique with varying approaches for intraocular lens implantation based on capsular support and zonular integrity. The most commonly performed procedure was phacoemulsification with foldable posterior chamber intraocular lens implantation in 49 patients (79.03%), followed by phacoemulsification with three-piece or PMMA IOL in ciliary sulcus in 8 patients (12.90%), phacoemulsification with secondary anterior chamber intraocular lens placement in 3 patients (4.83%) and phacoemulsification without IOL implantation in 2 patients (3.23%), as detailed in Table 2 (see PDF).

Intraoperative complications occurred in 11 patients (17.74%), with posterior capsular rent with or without vitreous loss being the most common complication in 7 patients (11.29%), followed by capsulorrhexis runout in 3 patients (4.83%) and intraoperative bleeding in 1 patient (1.61%). Despite these complications, surgical procedures were completed successfully in all cases with appropriate management of complications. Postoperative visual acuity assessment at 60 days follow-up demonstrated significant improvement in vision across all patients. A total of 19 patients (30.64%) achieved 6/6 vision, 15 patients (24.19%) achieved 6/9 vision, 12 patients (19.35%) achieved 6/12 vision, 9 patients (14.52%) achieved 6/18 vision, 4 patients (6.45%) achieved 6/24 vision and only 3 patients (4.83%) had vision worse than 6/24. Notably, 55 patients (88.70%) achieved vision of 6/18 or better, indicating successful visual rehabilitation in the vast majority of cases (Table 3 - see PDF). Postoperative complications were observed in 18 patients (29.04%), while 44 patients (70.96%) had an uneventful recovery. Cystoid macular edema was the most common complication, occurring in 8 patients (12.90%), followed by increased intraocular pressure in 5 patients (8.06%), IOL decentration in 2 patients (3.22%), posterior capsule opacification in 2 patients (3.22%) and optic capture in 1 patient (1.61%). Despite these complications, they were effectively managed with medical or surgical intervention, allowing most patients to maintain good visual outcomes. Comparative analysis of preoperative and postoperative visual acuity revealed remarkable improvement across all categories of initial visual impairment. Among patients with counting fingers vision preoperatively, 7 achieved 6/6 vision and 7 achieved 6/9 vision postoperatively. Similarly, patients with 6/60 vision showed substantial improvement, with 9 achieving 6/6 vision and 5 achieving 6/9 vision. Even patients with better preoperative vision demonstrated further improvement, with the majority achieving 6/6 or 6/9 vision postoperatively.

## Discussion:

This prospective study evaluated the visual and surgical outcomes of traumatic cataract surgery in 62 patients at a tertiary care center in Jharkhand, providing valuable insights into the management of this challenging condition in a resource-limited setting. The mean age of 39.5 years highlights that traumatic cataracts predominantly affect individuals in their productive years, leading to significant socioeconomic implications beyond visual impairment [10]. The male predominance of 70% aligns with previous studies and reflects the higher risk of occupational and sports-related ocular trauma in men [11]. The equal distribution of right and left eye involvement suggests that traumatic cataracts occur randomly with respect to laterality, without predilection for either eye. The higher prevalence of blunt trauma compared to penetrating trauma (59.67% vs 40.32%) indicates that workplace accidents, falls and similar mechanisms causing blunt force injuries are the predominant causes of traumatic cataracts in this population. This finding is consistent with the occupational profile of Jharkhand's population, where manual labor, mining and industrial activities are common [12]. The severity of preoperative visual impairment, with 71% of patients having vision of 6/60 or worse, underscores the significant impact of traumatic cataracts on daily functioning and quality of life. The high proportion of patients with counting fingers vision (30.64%) emphasizes the urgent need for surgical intervention to restore functional vision. These findings are comparable to other studies from similar settings, where traumatic cataracts present with severe visual impairment [13]. The exclusive use of phacoemulsification in this study reflects the advantages of this technique, including smaller incision size, faster recovery and better postoperative visual outcomes. The high success rate of posterior chamber IOL implantation (79.03%) indicates that despite trauma-related complications, adequate capsular support was maintained in most cases. The need for alternative IOL placement techniques in 20.97% of cases highlights the complexity of traumatic cataract surgery and the importance of surgical flexibility [14]. Intraoperative complications occurred in 17.74% of cases, with posterior capsular rent being the most common. This complication rate is within acceptable limits for traumatic cataract surgery, which is inherently more challenging than routine cataract surgery due to zonular instability and capsular weakness. The successful management of these complications without compromising final visual outcomes demonstrates the expertise required for traumatic cataract surgery [15]. The postoperative visual outcomes were excellent, with 88.70% of patients achieving vision of 6/18 or better at 60 days follow-up. This success rate is comparable to or better than many published studies on traumatic cataract surgery [16]. The achievement of 6/6 vision in 30.64% of patients and 6/9 vision in 24.19% of patients indicates that excellent visual rehabilitation is possible even in complex traumatic cases. The small percentage of patients with poor final vision (4.83%) likely reflects the severity of associated ocular trauma that could not be addressed through cataract surgery alone. The postoperative complication rate of 29.04% is reasonable for traumatic cataract surgery, considering the complexity of these cases. Cystoid macular edema, the most common complication at 12.90%, is a well-recognized sequela of intraocular surgery and responds well to anti-inflammatory treatment [17]. The occurrence of increased intraocular pressure in 8.06% of patients may be related to inflammatory response or retained lens material, both of which are manageable with appropriate medical therapy. The comparative analysis of preoperative and postoperative visual acuity demonstrates the transformative impact of surgical intervention. Patients with the poorest preoperative vision showed the most dramatic improvement, with many achieving excellent final vision. This finding supports the recommendation for early surgical intervention in traumatic cataract cases, regardless of initial visual acuity [18]. The study's findings have important implications for clinical practice and health policy. The high success rate of traumatic cataract surgery supports the establishment of specialized trauma centers and training programs for ophthalmic surgeons. The predominance of occupational trauma suggests the need for workplace safety measures and protective equipment to prevent ocular injuries [19]. Early referral and treatment protocols should be established to optimize outcomes, as delayed intervention may lead to secondary complications and poorer visual prognosis. Limitations of this study include the relatively short follow-up period of 60 days, which may not capture long-term complications or visual stability. The single-center design and specific population characteristics may limit generalizability to other settings. Future studies should include longer follow-up periods, multi-center designs and cost-effectiveness analyses to provide more comprehensive evidence for traumatic cataract management strategies. The study contributes valuable data to the limited literature on traumatic cataract outcomes in resource-limited settings. The excellent visual outcomes achieved through phacoemulsification demonstrate that with appropriate surgical expertise and patient selection, traumatic cataract surgery can be highly successful. The findings support the continued development of specialized ophthalmic services and training programs to address the significant burden of traumatic cataracts in regions with high occupational trauma rates [20]. Early surgical management of traumatic cataracts is associated with favorable visual outcomes; however, the presence of concomitant ocular injuries and postoperative complications significantly influences the final prognosis [21].

## Conclusion:

Cataract surgery in traumatic cases provides substantial visual rehabilitation, with most patients regaining functional vision when treated promptly. Phacoemulsification remains an effective and safe surgical option and postoperative complications are generally manageable with appropriate care. Strengthening early intervention, surgical expertise and preventive ocular safety measures can greatly improve outcomes and reduce the burden of traumatic cataracts in resource-limited settings.
